# Behavioral decision-making of the government, farmer-specialized cooperatives, and farmers regarding the quality and safety of agricultural products

**DOI:** 10.3389/fpubh.2022.920936

**Published:** 2022-10-05

**Authors:** Yun Teng, Boyuan Pang, Jingbo Wei, Li Ma, Huihui Yang, Zhanwei Tian

**Affiliations:** ^1^Northeast Agricultural University Engineering College, Harbin, China; ^2^Postdoctoral Mobile Station of Agricultural and Forestry Economic Management, Northeast Agricultural University, Harbin, China

**Keywords:** green production, agricultural product quality and safety, game evolution, farmer-specialized cooperatives, public health

## Abstract

The quality and safety of agricultural products is very important for farmers' professional cooperatives. This study incorporates the government, farmers' professional cooperatives and farmers into the evolutionary game model to explore the game relationship and evolutionary path of decision-making among the three parties related to the quality and safety of agricultural products. Through the dynamic analysis of decision-making replication, the analysis of strategy evolution stability and the verification of numerical simulation experiments, it is shown that the decision-making behavior of the government, farmers' professional cooperatives and farmers under the conditions of agricultural product quality and safety is the result of the game between three stakeholders; the government, farmers' professional cooperatives and farmers The evolution process of the decision-making behavior to the ideal state is affected by many factors, and the value ranges of different factors have different effects on the convergence speed of the three stakeholders to the ideal state; when certain conditions are met, the government, farmers' professional cooperatives and farmers' three The decision-making behavior of each stakeholder can evolve into an ideal state, and effective government supervision can promote the cooperatives to manage the green production of farmers, and then effectively encourage farmers to take the initiative in green production. In order to provide useful suggestions for the government to make safety supervision decisions, to effectively manage cooperatives, and to stimulate active green production behaviors by farmers.

## Introduction

The quality and safety of agricultural products is a major strategic issue related to China's economic development and social stability (1). Since the reform and opening up, as China's social and economic structure have gradually transformed, food safety issues have received increasing attention from the people. In recent years, incidents such as poisonous cowpea, poisonous ginger, strawberry pesticides exceeding the standard, Jiujiang “cadmium rice” and other incidents have made the quality and safety of agricultural products a common concern (2–5). The “National Quality Agriculture Strategic Plan (2018–2022)” pointed out the necessity of speeding the implementation of chemical pesticide reduction and substitution plans; supporting agricultural producers in the use of high-efficiency, low-toxicity and low-residue agricultural and veterinary drugs; and improving the quality and safety of agricultural products (6, 7).

Agricultural is an indispensably public healthcare industry for human beings at any time (8). The survey report released by the World Health Organization confirmed that the problem of agricultural product quality and safety is very common in the world. In the 19th century, foreign countries began to pay attention to the quality and safety of agricultural products. Upon Sinllair's “THE JUNGLE” recorded in detail the unsafe factors in Chicago's meat from breeding, production, and sales, and was the first to promote the development of agricultural product quality and safety. A large number of scholars have carried out extensive research on the quality and safety of agricultural products. These scholars are mainly from the perspective of food science and technology. Improve the quality and safety of agricultural products by cultivating improved varieties of agricultural products, improving the soil environment, and exploring production technologies such as pollution-free, green, or organic agricultural products (9–11). Other scholars from the perspective of economics, psychology, behavior, and other disciplines. Study the causes and solutions of agricultural product quality and safety problems. This includes the external government supervision system that studies the quality and safety of agricultural products from the macro level. The relationship between farmers' green production behavior and the quality and safety of agricultural products is studied from the perspective of micro-subject behavior (12–14).

The production behavior of farmers is the source that determines the quality and safety of agricultural products. The fundamental problem of agricultural product quality and safety is pollution at the source. Excessive application of pesticides and fertilizers has led to a serious problem of their residues in agricultural products. Arabian et al. (15) pointed out that farmers may suffer from cancer due to exposure to excessive pesticides (15). Agricultural products applied with excessive pesticides will also affect the health of end consumers. Many scholars have found that the nongreen production behaviors of farmers not only affect agricultural production but also cause great damage to consumer health. People's long-term consumption of inferior agricultural products leads to chronic poisoning and increase the incidence of cardiovascular, cerebrovascular diseases, Parkinson's disease, senile dementia and other diseases. Scholars believe that to ensure the quality and safety of agricultural products, we must start from the production source to avoid inferior agricultural products appearing on the table and affecting the physical and mental health of consumers (16–20). Therefore, it is of great significance to standardize, guide, and encourage the production behaviors of farmers to improve the quality and safety level of agricultural products.

Peasant-specialized cooperatives play an important role in intervening and preventing farmers' behaviors. The intervention of cooperatives on farmers' production behaviors mainly includes three intervention aspects: result, process, and social. Result intervention for cooperatives on farmers primarily consists of the unified testing and quality grading of agricultural products produced by farmers to restrain farmers' production behaviors (21, 22). Process intervention is mainly related to the unity of the peasant household production process management and supervision of farmers before, during and after the production process. such as formulating a series of production standards and stipulating the amount of pesticides and fertilizers applied by farmers can reduce the capital investment of farmers in the production process to a certain extent, and attracts more farmers to participate in it (23, 24). Social intervention means that cooperatives improve farmers' knowledge of agricultural green production and green production skills through rewards and punishments, archives and records, mutual cooperative supervision and technical training. It can promote the popularization, promotion and application of new agricultural scientific and technological achievements (25–27). Therefore, once an advanced cooperative management culture is formed, it can arouse farmers' strong interest in learning; improve their production skills and overall quality; mobilize the initiative, enthusiasm and creativity of farmers; and motivate them to implement green production behaviors (28).

Evolutionary game theory is an effective method for studying multiagent behavioral decision-making on the quality and safety of agricultural products. The existing research on multiagent behavioral decision-making in agricultural product quality and safety mostly focuses on bilateral games, and the study of tripartite games is less common. Cui et al. (29) constructed an evolutionary game model between the government and farmers and between farmers and agribusiness and established the optimal and stable strategy of green technology diffusion (29). The results show that the diffusion of inefficient technologies on both sides plays an important role in reducing the cost of green production and improving farmer income. Dang (30) makes a dynamic analysis of the evolution of the game between farmers, government and consumers (30). The results show that motivating consumers to participate in the market is conducive to maintaining market stability and controlling the quality of agricultural products. Liu et al. (31) established a game model among agricultural enterprises, the government and farmers (31). Their results showed that the reasonable subsidies and carbon tax can increase the enthusiasm of agricultural enterprises and farmers to participate in green and low-carbon agriculture to promote the production of green agricultural products. Du et al. (32) built a green agricultural production evolutionary game model under the market and government guidance mechanism (32). The results showed that the interaction between the main body determines the stability of the green agricultural production network and the quality and safety of agricultural products. Xu et al. (33) built a tripartite evolutionary game model, analyzed the evolutionary cooperation stability strategy, and discussed the interactive decision-making relationship between new agricultural operators and traditional farmers under the guidance of local governments (33). The results showed that new agricultural operators played a leading role in agricultural nonpoint source pollution control and a decisive role in maintaining the quality and safety of agricultural products. In this literature, the preliminary studies have mostly focused on the impact of the game between government and enterprises, cooperatives and consumers on the quality and safety of agricultural products. In later studies, the proportion of tripartite games gradually increased, but there were still few studies related to cooperatives, and games with individual farmers were lacking (34, 35). Therefore, this research includes the government, cooperatives and individual farmers in the same evolutionary game model. This paper builds a game model of dynamic evolution to reveal the safe production of the government, farmer-specialized cooperatives and individual farmer evolutionary paths and the evolutionary law of decision-making behaviour (36, 37). It is expected to provide reference for the government to improve the efficiency of supervision, for cooperatives to better regulate the behavior of farmers, and for farmers to take the initiative to carry out green production and maintain the quality and safety of agricultural products.

## Hypothesis and model construction

The behaviors of government, farmer-specialized cooperatives and individual farmers are incorporated into a game system, and the three stakeholders all have the characteristics of bounded rationality and the ability to learn and imitate.

The profit and loss analysis of the relevant stakeholders with different strategies is as follows:

Government-related gains and losses are as follows: Assuming that the probability of the government taking safety regulatory measures is x (0 ≦ x ≦ 1), then the probability of not taking safety regulatory measures is (1 − x).

When the government supervises the safety of farmer-specialized cooperatives, the cost of supervision is *C*_1_, the beneficial impact of the government's access to cooperatives to manage farmers' safe production to the government is *D*_1_, the government's safety supervision being conducive to social stability is *W*_1_, the fine paid by cooperatives that do not manage the safe production of farmers is *M*_1_, and the bonus for cooperatives that manage the safe production of farmers is *M*_2_.

When the government does not supervise the safety of farmer-specialized cooperative, the cost savings for safety regulation are *C*_1_, the governments' safety supervision having beneficial administrative effects is *D*_1_, the adverse effects of the government not managing cooperative farmers production safety is *D*_2_, and the negative effects on society of the government not regulating safety (including the quality and safety of agricultural products, farmers' own safety, consumer's physical and mental health, social stability, etc.) are *W*_2_.

Relevant profits and losses of farmer-specialized cooperatives are as follows: Suppose that the probability of farmer-specialized cooperatives managing farmer production behaviors is y, then the probability of nonmanagements is 1-y (0 ≦ y ≦ 1).

If farmer-specialized cooperatives affect the behaviors of peasant household production management, the cooperative management cost (including specification management cost, testing cost and training cost, etc.) is *C*_2_, the government gives farmers' cooperative management production safety bonuses of *M*_2_, the government fines farmers for not producing safely in the amount of *M*_3_, farmers pay dividends of *M*_4_ for safe production, safe production behavior is advantageous to the cooperative development of farmers *D*_3_, farmers produce high-quality agricultural products, and the promoted cooperative benefits are *B*_1_.

If the farmer-specialized cooperatives do not manage farmer production behaviors, a management cost of *C*_2_ can be saved; Because the cooperative did not manage the farmers, the fine to the government was *M*_1_. At the same time, the cooperative will also lose the bonus *M*_2_ given by the government, and the fine *M*_3_ paid by farmers for unsafe production; Save the dividend *M*_4_ given to safe production farmers; The unsafe production behavior of farmers has adversely affected the development of cooperatives as *D*_4_; the non-high-quality agricultural products produced by farmers are not high-quality and favorable prices, and the loss to the cooperative's income is *B*_2_.

The relevant profits and losses of farmers are as follows: Assuming that the proportion of farmers choosing green applications is z, then the proportion of farmers choosing nongreen applications is 1-z (0 ≦ z ≦ 1). The positive (negative) impact of government safety supervision on farmers is *T*_1_ (*T*_2_).

If a farmer carries out green applications, the cost of purchasing green agricultural materials is *C*_3_, the income increase from selling high-quality agricultural products is *S*_1_, the dividend from the cooperative for safe production is *M*_4_, and the beneficial impact (to the health of the farmer and the protection of land) of green application on farmers is *W*_3_.

If farmers choose to use green pesticides, the cost savings for green agricultural materials is *C*_3_, the unsold nongreen agricultural products are *S*_2_, the farmers income lost due to the green pesticide results in a need to pay a penalty to the cooperative farmers of *M*_3_, the damage the farmers cooperatives cause results in green pesticide dividends *M*_4_, and the farmers use of nongreen pesticide results in adverse effects (their health is impaired, the green pesticide destroys the land, etc.) of *W*_4_.

Therefore, the payoff matrix can be obtained for 8 strategy combination: whether the government adopts safety supervision measures, whether farmer-specialized cooperatives encourage members to carry out safe production, and whether farmers choose green applications. The specific contents are shown in Table 1.

**Table 1:** Revenue combination of tripartite evolutionary game.

**Strategy combination**	**Government revenue**	**Profits from specialized farmer cooperatives**	**Farmers income**
(Regulatory, management, green application)	−*C*_1_ + *D*_1_ + *W*_1_−*M*_2_	−*C*_2_ + *M*_2_−*M*_4_ + *D*_3_ + *B*_1_	*T*_1_−*C*_3_ + *S*_1_ + *M*_4_ + *W*_3_
(Regulatory, management, non-green application)	−*C*_1_ + *D*_1_ + *W*_1_−*M*_2_	−*C*_2_ + *M*_2_ + *M*_3_−*D*_4_−*B*_2_	*T*_1_ + *C*_3_−*S*_2_−*M*_3_−*W*_4_
(Regulatory, not manage, green application)	−*C*_1_ + *W*_1_ + *M*_1_	*C*_2_−*M*_1_ + *D*_3_ + *B*_1_	*T*_1_−*C*_3_ + *S*_1_ + *W*_3_
(Regulatory, not manage, non-green application)	−*C*_1_ + *W*_1_ + *M*_1_	*C*_2_−*M*_1_−*D*_4_−*B*_2_	*T*_1_ + *C*_3_−*S*_2_−*W*_4_
(Not regulate, management, green application)	*C*_1_ + *D*_1_−*W*_2_	−*C*_2_−*M*_4_ + *D*_3_ + *B*_1_	−*T*_2_−*C*_3_ + *S*_1_ + *M*_4_ + *W*_3_
(Not regulate, management, non-green application)	*C*_1_ + *D*_1_−*W*_2_	−*C*_2_ + *M*_3_−*D*_4_−*B*_2_	−*T*_2_ + *C*_3_−*S*_2_−*M*_3_−*W*_4_
(Not regulate, not manage, green application)	*C*_1_−*D*_2_−*W*_2_	*C*_2_ + *D*_3_ + *B*_1_	−*T*_2_−*C*_3_ + *S*_1_ + *W*_3_
(Not regulate, not manage, non-green application)	*C*_1_−*D*_2_−*W*_2_	*C*_2_−*D*_4_−*B*_2_	−*T*_2_ + *C*_3_−*S*_2_−*W*_4_

ESS in the table below is Evolutionary stability strategy.

In this relationship, according to the actual situation, constraint condition can be added as follows: If farmers carry out green applications, the increased income from producing high-quality agricultural products should be greater than the cost of purchasing agricultural materials, that is, *C*_3_<*S*_1_. If farmers apply nongreen agricultural materials, the cost of the green agricultural resources they save should be less than the loss caused by not being able to sell nongreen products at a high price, that is, *C*_3_<*S*+2.

## Analysis of the equilibrium of the evolutionary game between the government, farmer-specialized cooperatives and individual farmer

According to the income matrix in Table 1, the expected income *V*_1*x*_, *V*_2*x*_ and the average income $\bar{V_{x}}$ of the government departments' decision making on “safety supervision” and “no safety supervision” can be obtained. The expected income *V*_1*y*_, *V*_2*y*_,.. and average income $\bar{V_{y}}$ of the decision-making of “management of farmers' production behavior” and “non-management of farmers' production behavior” of farmer-specialized cooperatives. And the expected income *V*_1*z*_, *V*_2*z*_ and average income $\bar{V_{z}}$ of the farmers choosing “green application” and “non-green application.” The specific formula is shown in the Appendix.

### Replication dynamic analysis of government safety supervision decisions

According to the expected benefits corresponding to different decisions of government departments, the dynamic analysis equation of government safety supervision behavior decision replication is derived:


(1) $$\begin{array}{l} F\left(x\right)\;=\;\frac{dx}{dt}\;=\;x\left(V_{1x}\;-\;\bar{V_{x}}\right)\\ \;=\;x\left(1\;-\;x\right)\left[\begin{array}{l} -2C_{1}\;+\;D_{1}\;+\;M_{1}\;+\;W_{1}\;+\;W_{2}\\ -y\left(D_{1}\;+\;M_{1}\;+\;M_{2}\right) \end{array}\right] \end{array}$$

When $y\;=\;\frac{-2C_{1}\;+\;D_{1}\;+\;M_{1}\;+\;W_{1}\;+\;W_{2}}{D_{1}\;+\;M_{1}\;+\;M_{2}}$, *F*(*x*) = 0, which means that whether the government conducts safety supervision in a stable state.

When $y\neq \frac{-2C_{1}\;+\;D_{1}\;+\;M_{1}\;+\;W_{1}\;+\;W_{2}}{D_{1}\;+\;M_{1}\;+\;M_{2}}$, set *F*(*x*) = 0 to obtain *x* = 0, and *x* = 1 may be stable points. According to the stability theorem of the replicated dynamic equation, *x*, as a stable strategy, needs to meet *F*(*x*) = 0 and *F*′(*x*) < 0. Taking the derivative of *F*(*x*):


(2) $$F^{\prime }\left(x\right)\;=\;\left(1\;-\;2x\right)\left[\begin{array}{l} -2C_{1}\;+\;D_{1}\;+\;M_{1}\;+\;W_{1}\;+\;W_{2}\\ -y\left(D_{1}\;+\;M_{1}\;+\;M_{2}\right) \end{array}\right]$$

When $y>\frac{-2C_{1}\;+\;D_{1}\;+\;M_{1}\;+\;W_{1}\;+\;W_{2}}{D_{1}\;+\;M_{1}\;+\;M_{2}}$, ${\frac{dF\left(x\right)}{dx}|}_{x\;=\;0}<0$, and ${\frac{dF\left(x\right)}{dx}|}_{x\;=\;1}>0$; therefore, is the evolutionary stable point.

When $y<\frac{-2C_{1}\;+\;D_{1}\;+\;M_{1}\;+\;W_{1}\;+\;W_{2}}{D_{1}\;+\;M_{1}\;+\;M_{2}}$, and ${\frac{dF\left(x\right)}{dx}|}_{x\;=\;1}<0$; therefore, *x* = 1 is the evolutionary stable point.

### Replicative dynamic analysis of farmers' production decision-making in farmer-specialized cooperatives

According to the expected benefits corresponding to different decisions of farmers' professional cooperatives, the dynamic analysis equation for copying production decisions of farmer-specialized cooperative management of farmers' production is derived:


(3) $$\begin{array}{l} F\left(y\right)\;=\;\frac{dy}{dt}\;=\;y\left(V_{1y}\;-\;\bar{V_{x}}\right)\\ F^{\prime }\left(y\right)=\left(1\;-\;y\right)\left[\begin{array}{l} x\left(M_{1}\;+\;M_{2}\right)\\ -z\left(M_{3}\;+\;M_{4}\right)\\ -2C_{2}\;+\;M_{3} \end{array}\right] \end{array}$$

When $x\;=\;\frac{z\left(M_{3}\;+\;M_{4}\right)\;+\;2C_{2}-M_{3}}{M_{1}\;+\;M_{2}}$, *F*(*y*) = 0, which means that whether farmer-specialized cooperatives manage farmers in carrying out safe production is in a stable state.

When $x\neq \frac{z\left(M_{3}\;+\;M_{4}\right)\;+\;2C_{2}-M_{3}}{M_{1}\;+\;M_{2}}$, set *F*(*y*) = 0 to obtain *y* = 0, and *y* = 1 can all be stable points. According to the stability theorem of the replicated dynamic equation, x, as a stable strategy, needs to conform to *F*(*y*) = 0 and *F*′(*y*) < 0. The derivative of *F*(*y*) is:


(4) $$F^{\prime }\left(y\right)\;=\;\left(1-2y\right)\left[\begin{array}{l} x\left(M_{1}\;+\;M_{2}\right)\\ -z\left(M_{3}\;+\;M_{4}\right)\\ -2C_{2}\;+\;M_{3} \end{array}\right]$$

When $x\;>\;\frac{z\left(M_{3}\;+\;M_{4}\right)\;+\;2C_{2}-M_{3}}{M_{1}\;+\;M_{2}}$, ${\frac{dF\left(y\right)}{dy}|}_{y\;=\;0}>0$, and ${\frac{dF\left(y\right)}{dy}|}_{y\;=\;1}<0$; therefore, *y* = 1 is the evolutionary stable point.

When $x<\frac{z\left(M_{3}\;+\;M_{4}\right)\;+\;2C_{2}-M_{3}}{M_{1}\;+\;M_{2}}$, ${\frac{dF\left(y\right)}{dy}|}_{y\;=\;0}<0$, and ${\frac{dF\left(y\right)}{dy}|}_{y\;=\;1}>0$; therefore, *y* = 0 is the evolutionary stable point.

### Replicative dynamic analysis of farmers' decision-making for safe production behaviors

According to the expected income corresponding to different decisions of farmers, the dynamic analysis equation of farmers' safe production behavior decision replication is derived:

When $y\;=\;\frac{2C_{3}-S_{1}-S_{2}-W_{3}-W_{4}}{M_{3}\;+\;M_{4}}$, *F*(*z*) = 0, which means that the farmers; safe production is in a stable state.

When $y\neq \frac{2C_{3}-S_{1}-S_{2}-W_{3}-W_{4}}{M_{3}\;+\;M_{4}}$, set *F*(*z*) = 0 to obtain *z* = 0, and *z* = 1 can all be stable points. According to the stability theorem of the replicated dynamic equation, *z*, as a stability strategy, needs to conform to *F*(*z*) = 0 and *F*′(*z*) < 0. The derivative of *F*(*z*) is obtained as follows:

When, ${\frac{dF\left(z\right)}{dz}|}_{z\;=\;0}<0$, and ${\frac{dF\left(z\right)}{dz}|}_{z\;=\;1}>0$; therefore, *z* = 0 is the evolutionary stable point.

When, ${\frac{dF\left(z\right)}{dz}|}_{z\;=\;0}>0$, and ${\frac{dF\left(z\right)}{dz}|}_{z\;=\;1}<0$; therefore, *z* = 1 is the evolutionary stable point.

### Replicative dynamic analysis of farmers' decision-making for safe production behaviors

Equations (1), (3) and (5) indicate that government safety supervision decisions are related to farmers' production decisions managed by farmer-specialized cooperatives, the production decisions of farmers under cooperative management are related to the government's safety supervision decisions and farmers' safety production decisions, and the decision-making of farmers' safe behaviors is related to the decision-making of farmers' production in cooperative management. Therefore, this study conducts a stepwise analysis of the strategic evolutionary stability of the government, farmer-specialized cooperatives and individual farmers, namely, evolutionary stability analyses of the government and farmer-specialized cooperatives and of individual farmers and farmer-specialized cooperatives.

#### Analysis of the evolutionary stability of the government and farmer-specialized cooperatives

According to Equations (1) and (5), the dynamic game between government and cooperatives contains five equilibrium points (0, 0), (0, 1), (1, 0), (1, 1), and ($x^{*}\;=\;\frac{z\left(M_{3}\;+\;M_{4}\right)\;+\;2C_{2}\;-\;M_{3}}{M_{1}\;+\;M_{2}}$, $y^{*}\;=\;\frac{-2C_{1}\;+\;D_{1}\;+\;M_{1}\;+\;W_{1}\;+\;W_{2}}{D_{1}\;+\;M_{1}\;+\;M_{2}}$). If and only if $0\leq \frac{z\left(M_{3}\;+\;M_{4}\right)\;+\;2C_{2}-M_{3}}{M_{1}\;+\;M_{2}}\leq 1$, $0\leq \frac{-2C_{1}\;+\;D_{1}\;+\;M_{1}\;+\;W_{1}\;+\;W_{2}}{D_{1}\;+\;M_{1}\;+\;M_{2}}\leq 1$, the dynamic game evolution is given.

Jacobi matrix:


$$\begin{array}{l} J1\;=\;\left[\begin{array}{cc} \left(1\;-\;2x\right)\left[\begin{array}{c} -2C_{1}\;+\;D_{1}\\ \;+\;M_{1}\;+\;W_{1}\;+\;W_{2}\\ -y\left(D_{1}\;+\;M_{1}\;+\;M_{2}\right) \end{array}\right], & x\left(1\;-\;x\right)\left(D_{1}\;+\;M_{1}\;+\;M_{2}\right)\\ y\left(1\;-\;y\right)\left(M_{1}\;+\;M_{2}\right), & \left(1\;-\;2y\right)\left[\begin{array}{c} x\left(M_{1}\;+\;M_{2}\right)\\ -z\left(M_{3}\;+\;M_{4}\right)\\ -2C_{2}\;+\;M_{3} \end{array}\right] \end{array}\right] \end{array}$$

The determinant of matrix *J*1:


$$\begin{array}{l} \det J1\;=\;\left(1\;-\;2x\right)\left[\begin{array}{l} -2C_{1}\;+\;D_{1}\;+\;M_{1}\;+\;W_{1}\;+\;W_{2}\\ -y\left(D_{1}\;+\;M_{1}\;+\;M_{2}\right) \end{array}\right]\\ \;*\left(1\;-\;2y\right)[x\left(M_{1}\;+\;M_{2}\right)\;-\;z\left(M_{3}\;+\;M_{4}\right)-2C_{2}\\ \;+M_{3}]-x\left(1\;-\;x\right)\left(D_{1}\;+\;M_{1}\;+\;M_{2}\right)y\left(1\;-\;y\right)\\ \;\left(M_{1}\;+\;M_{2}\right) \end{array}$$

Matrix *J*1 trace:


$$\begin{array}{l} \det J1\;=\;\left(1\;-\;2x\right)\left[\begin{array}{l} -2C_{1}\;+\;D_{1}\;+\;M_{1}\;+\;W_{1}\;+\;W_{2}\\ -y\left(D_{1}\;+\;M_{1}\;+\;M_{2}\right) \end{array}\right]\\ \;*\left(1\;-\;2y\right)[x\left(M_{1}\;+\;M_{2}\right)\;-\;z\left(M_{3}\;+\;M_{4}\right)-2C_{2}\\ \;+M_{3}]-x\left(1\;-\;x\right)\left(D_{1}\;+\;M_{1}\;+\;M_{2}\right)y\left(1\;-\;y\right)\\ \;\left(M_{1}\;+\;M_{2}\right) \end{array}$$

$$J2\;=\;\left[\begin{array}{l} \left(1\;-\;2y\right)\left[\begin{array}{l} x\left(M_{1}\;+\;M_{2}\right)\\ -z\left(M_{3}\;+\;M_{4}\right)\\ -2C_{2}\;+\;M_{3} \end{array}\right],\;2y\left(1\;-\;y\right)\left(M_{3}\;+\;M_{4}\right)\;\\ \;z\left(1\;-\;z\right)\left(M_{3}\;+\;M_{4}\right),\;\left(1\;-\;2z\right)\left[\begin{array}{l} S_{1}\;+\;S_{2}\;+\;W_{3}\;+\;W_{4}\\ -2C_{3}\;+\;y\left(M_{3}\;+\;M_{4}\right) \end{array}\right] \end{array}\right]$$

A local stability analysis was conducted according to the above five equilibrium points, and the results are shown in Table 2.

**Table 2:** Stability analysis of the evolutionary game of government farmer specialized cooperatives.

**Equilibrium**	**detJ_1_ symbol**	**trJ_1_ symbol**	**Result**	**Stability condition**
*x* = 0, *y* = 0	-	Not sure	Saddle point	It's a saddle point under any condition
*x* = 0, *y* = 1	-	Not sure	Saddle point	It's a saddle point under any condition
*x* = 1, *y* = 0	-	Not sure	Saddle point	It's a saddle point under any condition
*x* = 1, *y* = 1	+	-	ESS	*W*_1_ + *W*_2_−*M*_2_>2*C*_1_, *M*_1_ + *M*_2_ + *M*_3_>2*C*_3_ + *z*(*M*_3_ + *M*_4_)
*x* = x^*^, *y* = y^*^	0	0	Saddle point	It's a saddle point under any condition

Table 2 indicates that stability points can be formed in the dynamic evolutionary process of government and farmer-specialized cooperation if the following conditions are met:

Whether the government safety supervision measures for social stability and the influence of the government on managing the peasant household production cooperatives bonuses for a government safety regulation cost is >2 times the difference. Moreover, the government manages farmer-specialized cooperatives not by paying fines but by giving bonuses, and cooperatives never pay farmers fines, the result of which is greater than the sum of twice the production safety management cost. Finally, the farmer-specialized cooperatives are not penalized, and the sum is that of the mathematical expectation of the share-out bonus. Then, the result of the game between the government and farmer-specialized cooperatives is the stable state *x* = 1, *y* = 1, that is, the government safety supervision and farmers' safe production behaviors.

#### Analysis of the evolutionary stability of the government and farmer-specialized cooperatives

According to Equations (1) and (3), the dynamic game between peasant households and farmer-specialized cooperatives contains five equilibrium points (0,0), (0,1), (1,0), (1,1), and $(y^{**}\;=\;\frac{2C_{3}\;-\;S_{1}\;-\;S_{2}\;-\;W_{3}\;-\;W_{4}}{M_{3}\;+\;M_{4}}$, $z^{**}\;=\;\frac{x\left(M_{1}\;+\;M_{2}\right)\;-\;2C_{2}\;+\;M_{3}}{\left(M_{3}\;+\;M_{4}\right)})$. If and only if $0\leq \frac{2C_{3}\;-\;S_{1}\;-\;S_{2}\;-\;W_{3}\;-\;W_{4}}{M_{3}\;+\;M_{4}}\leq 1$, $0\leq \frac{x\left(M_{1}\;+\;M_{2}\right)-2C_{2}\;+\;M_{3}}{\left(M_{3}\;+\;M_{4}\right)}\leq 1$, the dynamic game evolution is given.

Jacobi matrix:

The determinant of matrix*J*2:


$$\begin{array}{l} \det J2\;=\;\left(1\;-\;2y\right)[x\left(M_{1}\;+\;M_{2}\right)-z\left(M_{3}\;+\;M_{4}\right)-2C_{2}\\ \;+M_{3}]\left(1\;-\;s2z\right)[S_{1}\;+\;S_{2}\;+\;W_{3}\;+\;W_{4}-2C_{3}\\ \;+\;y\left(M_{3}\;+\;M_{4}\right)]\;-\;2y\left(1\;-\;y\right)\left(M_{3}\;+\;M_{4}\right)\\ \;z\left(1\;-\;z\right)\left(M_{3}\;+\;M_{4}\right) \end{array}$$

Matrix trace:


$$\begin{array}{l} trJ2\;=\;\left(1\;-\;2y\right)[x\left(M_{1}\;+\;M_{2}\right)\;-\;z\left(M_{3}\;+\;M_{4}\right)-2C_{2}\\ \;+M_{3}]\;+\;\left(1-2z\right)[S_{1}\;+\;S_{2}\;+\;W_{3}\;+\;W_{4}-2C_{3}\\ \;+\;y\left(M_{3}\;+\;2M_{4}\right)] \end{array}$$

A local stability analysis was conducted according to the above five equilibrium points, and the results are shown in Table 3.

**Table 3:** Stability analysis of evolutionary game of peasant households and specialized farmer cooperatives.

**Equilibrium**	**detJ_2_ symbol**	**trJ_2_ symbol**	**Result**	**Stability condition**
*y* = 0, *z* = 0	-	Not sure	Saddle point	It's a saddle point under any condition
*y* = 0, *z* = 1	+	-	ESS	*x*(*M*_1_ + *M*_2_) < *M*_4_ + 2*C*_2_ *S*_1_ + *S*_2_ + *W*_3_ + *W*_4_>2*C*_3_
y = 0,z = 1	−	Not sure	Saddle point	It's a saddle point under any condition
y = 1, z = 0	+	−	ESS	*x*(*M*_1_ + *M*_2_) > *M*_4_ + 2*C*_2_ *S*_1_ + *S*_2_ + *W*_3_ + *W*_4_ + *M*_3_ + *M*_4_ > 2*C*_3_
y = 1, z = 1	0	0	Saddle point	It's a saddle point under any condition

Table 3 indicates that stable points can be formed in the dynamic evolutionary process of peasant households and farmer-specialized cooperatives if the following conditions are met:

When the government manages production cooperatives, farmers pay fines and give farmers production cooperatives the mathematical expectation of the sum of the bonuses of < 2 times the cooperative management costs and cooperatives give the sum of the farmers production safety share-out bonuses. Moreover, farmers selling high-quality agricultural products experience increased revenue and use more green pesticide. The beneficial effects of the green agricultural products could result in high losses amounts of lost farmers income. Additionally, the negative influence of the farmers use of green pesticide is greater than the sum of twice the farmers purchase of green agricultural capital. Then, the result of the game between peasant households and Peasant-specialized cooperatives is in stable state *y* = 0, *z* = 1; that is, peasant households produce safely, and cooperatives do not manage peasant households' production.

When the government's mathematical expectation that the sum of the fines paid by the government for cooperatives that do not manage farmer's production and the bonuses given to the cooperatives that manage farmer's production is less than twice the management cost of the cooperative and the sum of the dividends paid by the cooperative to farmers for safe production, and the sum of the increased income of farmers selling high-quality agricultural products and the beneficial effects of green pesticides on farmers, and the loss of income of farmers due to the inability to sell non-green agricultural products at a high price, and the adverse effects of non-green pesticides on farmers more than double the cost of farmers buying green agricultural materials. The result of the game between peasant households and Peasant-specialized cooperatives is the stable state *y* = 1, *z* = 1; that is, peasant households produce safely and Peasant-specialized cooperative manage peasant households' production.

## Numerical experiment and simulation

Starting from the idea of people-oriented management, this study promotes the ultimate evolution of the tripartite game between the government, farmer-specialized cooperatives and individual farmers to the ideal decision-making state of government safety supervision, farmer-specialized cooperatives managing farmers' safe production and individual farmers' safe production (*x* = 1, *y* = 1, *z* = 1). This study applied the constraint conditions and the replication of the dynamic equation using the national statistical yearbook published data, a multisubject questionnaire, a field investigation, and an analysis of three ways to obtain data and data preprocessing. This study used MATLAB simulation software to analyse the government, farmer-specialized cooperatives, and individual farmers tripartite game ideal state numerical test. Set the parameter values according to the constraint conditions, which were as follows:*S*_1_ = 20, *S*_2_ = 25, *M*_1_ = 60, *M*_2_ = 50, *M*_3_ = 20, *M*_4_ = 10, *C*_1_ = 40, *C*_2_ = 20, *C*_3_ = 10, *D*_1_ = 15, *D*_2_ = 20, *W*_1_ = 70, *W*_2_ = 80, *W*_3_ = 20, *W*_4_ = 30, *T*_1_ = 20, *T*_2_ = 25.

**Figure 1:**
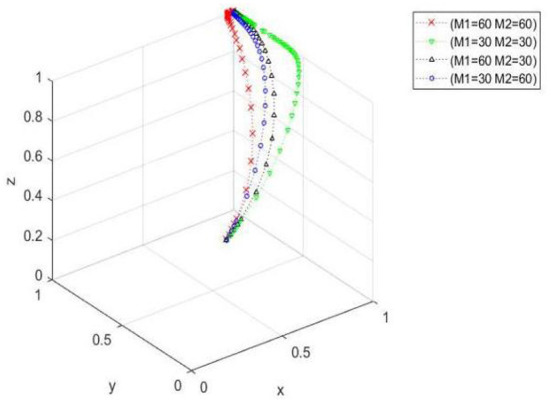
Spatial diagram of the influence of changes in incentive and punishment on the evolutionary paths of cooperatives given by the government.

The influence of government punishment and reward on the cooperative evolution. When *x*_0_ = 0.5, *y*_0_ = 0.4, and, the parameter values are *M*_1_ = 60, *M*_2_ = 60; *M*_1_ = 30, *M*_2_ = 30; *M*_1_ = 60, *M*_2_ = 30; *M*_1_ = 30, and *M*_2_ = 60, respectively. Figure 1 shows that when the government rewards (punishes) the cooperatives that (do not) manage farmers' safe production, the ideal state of the three directions evolves. When the punishment is fixed, with an increasing reward, the convergence speed of the tripartite evolutionary system increases, and the time needed to converge to the ideal state decreases. When the reward is constant, the tripartite evolutionary system converges faster, and the time needed to converge to the ideal state decreases as the punishment increases. When the reward is less than the punishment, the convergence rate of the tripartite evolutionary system decreases, and the time needed to converge to the ideal state increases. When the reward is greater than the punishment, the tripartite evolutionary system converges faster, and the time needed to converge to the ideal state decreases. When the reward and punishment are both large, the three-way evolutionary system converges the fastest and takes the least amount of time to converge to the ideal state.

**Figure 2:**
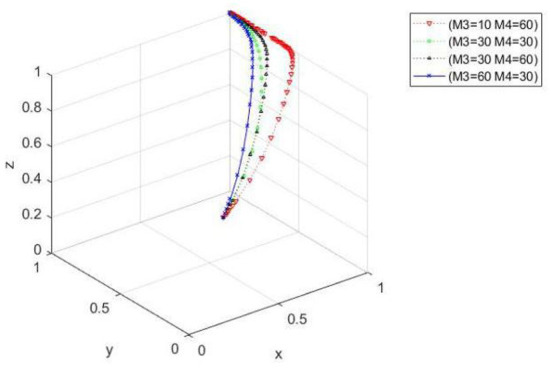
Spatial diagram of the influence of the change of incentive and punishment on the evolutionary path of farmers given by cooperatives.

Effects of rewards and punishment for green and nongreen drug application by farmer-specialized cooperatives on evolutionary processes. When *x*_0_ = 0.5, *y*_0_ = 0.4, and *z*_0_ = 0.3, the parameter values are *M*_3_ = 10,*M*_4_ = 60; *M*_3_ = 30, *M*_4_ = 30; *M*_3_ = 30, *M*_4_ = 60; *M*_3_ = 60, and *M*_4_ = 30, respectively. Figure 2 shows that when cooperatives reward (punish) farmers for green (nongreen) applications, the ideal state evolves in three directions. When the reward is less than the punishment, the convergence rate of the tripartite evolutionary system decreases, and the time needed to converge to the ideal state increases. When the reward is greater than the punishment, the tripartite evolutionary system converges faster, and the time needed to converge to the ideal state decreases. When both rewards and punishments are large, the tripartite evolutionary system converges the fastest and takes the least amount of time to converge to the ideal state.

**Figure 3:**
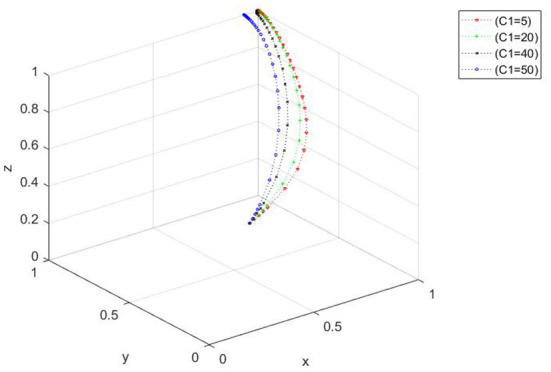
Spatial diagram of the impact of the change of government investment in safety supervision costs on the evolutionary path.

The influence of the change of the governmental regulation cost on the evolutionary process. When *x*_0_ = 0.5, *y*_0_ = 0.4, and *z*_0_ = 0.3, the *C*_1_ values are 5, 20, 40, and 50. Figure 3 shows that when the government supervision cost increases, the three-party evolutionary system converges faster, and the time needed to converge to the ideal state decreases. However, when the cost of government supervision gradually increases and exceeds a certain range, the evolutionary path changes, and the ideal state cannot be realized.

**Figure 4:**
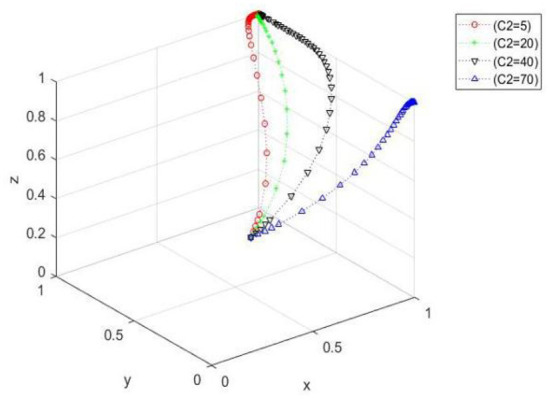
Spatial diagram of the influence of the change of production safety cost on the evolution path of peasant households managed by cooperatives.

The influence of the change of production safety cost on the evolutionary process of farmer-specialized cooperative management. When *x*_0_ = 0.5, *y*_0_ = 0.4, and *z*_0_ = 0.3, the *C*_2_ values are 5, 20, 40, and 70. Figure 4 shows that when the management cost gradually increases, the convergence speed of the three-party evolutionary system decreases, and the time needed to converge to the ideal state increases. However, when the management cost of cooperatives gradually increases beyond a certain range, the evolutionary path changes greatly, and cooperatives cannot attain to the ideal state.

**Figure 5:**
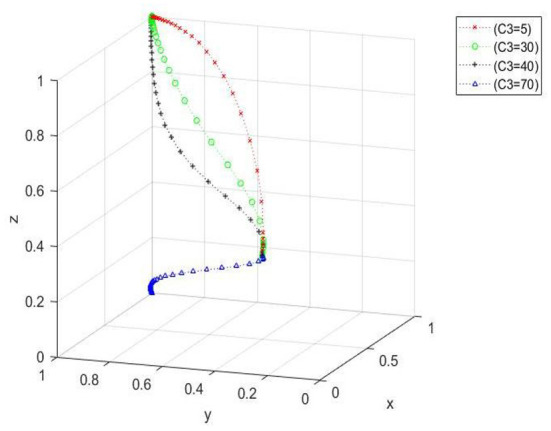
Spatial diagram of the impact of changes in the cost of purchasing green agricultural materials on the evolutionary path of farmers.

Impact of changes in farmers' cost of purchasing green agricultural materials on the evolutionary process. When *x*_0_ = 0.4, *y*_0_ = 0.6, and *z*_0_ = 0.5, the *C*_3_ values are 5, 30, 40, and 70. Figure 5 shows that when the purchase cost exceeds a certain range, the evolutionary path changes greatly, and the ideal state cannot be approached. When buying costs within the scope of the medium, as costs increase, the evolution of the tripartite system convergence increases, the time required to converge to the ideal state reduces, and an ideal state eventually evolves. However, the cost is large, the evolution of the tripartite system convergence speed decreases again, the time required to converge to an ideal state increases again, but ultimately an ideal state still evolves.

## Conclusion and countermeasures

### Research conclusions

This paper makes a dynamic analysis of the decision-making replication, evolutionary stability analysis and numerical simulation experiment verification among the three stakeholders of government, farmer-specialized cooperatives and farmer safety management. The main conclusions are as follows:

The decision-making replicated dynamic equation indicates that the proportion of farmers' professional cooperatives that manage farmers' production decisions is related to the proportion of government safety supervision decisions and the proportion of farmers' safety production decisions, the proportion of farmers' safety production decision-making is related to the proportion of farmers' production decision-making under the management of farmers' professional cooperatives. Specifically, government decisions are directly affected by cooperative decisions but are not be affected by farmers' decisions. The decisions of farmer-specialized cooperatives are influenced by both the government's and individual farmers' decisions. Individual farmers' decisions are influenced by cooperative decisions. The government, cooperatives and farmers bridge the decision-making process. In the process of production safety management, it is necessary to clarify the influence relationship among the three.

The analysis of evolutionary stability indicates that the ideal state of the three parties needs to meet four conditions at the same time:

The difference between the impact of safety supervision measures on social stability and the bonus given by the government to the cooperatives that manage farmers' production is more than 2 times the cost of government safety supervision.

The sum of the fines paid by the government to the cooperatives that do not manage the farmers, the bonuses they give to the cooperatives and the fines they pay to the farmers for unsafe production is greater than the sum of the mathematical expectation of the management cost of the cooperatives and the sum of the fines and dividends they pay to the farmers.

The mathematical expectation of the sum of the fine paid by the government to cooperatives that do not manage the production of peasant households and the bonus given by those cooperatives to cooperatives that manage the production of peasant households is greater than the sum of the management cost of the cooperative and the bonus given by the cooperative to the safe production of peasant households.

“The increased income of farmers from selling high-quality agricultural products and the beneficial effects of green pesticides on farmers,” “The loss of income from farmers who cannot sell non-green agricultural products at high prices,” “The adverse effects of non-green pesticides on farmers” and “The fines and dividends paid by cooperatives to farmers,” the sum of the above four is more than twice the cost of farmers' green agricultural materials. The government, farmer-specialized cooperatives and individual farmers can achieve the ideal state of safe production; that is, the government actively regulates safety, the cooperatives actively manage the production of farmers, and farmers carry out the green application of fertilizers.

The numerical simulation results indicate that the evolutionary process of the three to the ideal state is as follows:

The government adopts four reward and punishment modes: light reward and light punishment, heavy punishment and light reward, light punishment and heavy reward, heavy reward and heavy punishment. The convergence speed of the three party evolutionary system gradually accelerates, and the time needed for convergence to the ideal state gradually decreases.

Farmer-specialized cooperatives reward (punish) farmers for green (non-green) applications. When the reward is heavy and the punishment is light, the convergence rate of the evolutionary system of the three parties is slow, and it takes a long time to converge to the ideal state. When the reward is heavy and the punishment is heavy, the tripartite evolutionary system converges the fastest and takes the least amount of time to converge to the ideal state.

The cost of government supervision increases within a certain range, the convergence speed of the tripartite evolutionary system is accelerated, and the time needed to converge to the ideal state is reduced.

When the management cost of farmer-specialized cooperatives gradually increases, the convergence speed of the evolutionary system of the three parties decreases, and the time needed to converge to the ideal state increases. The management cost exceeds a certain range and is unable to trend to the ideal state.

The cost of purchasing green agricultural materials for farmers is too large or too small, and it takes a long time for them to reach the ideal state. However, if the cost is increased within an appropriate range, the time required for the three parties to converge to the ideal state will be reduced. However, the cost of purchasing green agricultural materials exceeds a certain range and cannot trend to a stable state.

### Implications

Using a “theoretical research—mathematical modeling—numerical simulation,” the paper reveals the characteristics and rules of the decision-making behaviors of the three stakeholders—the government, farmer-specialized cooperatives and individual farmers—and draws the following conclusions:

The government is a strong promoter of green production and encourages farmer-specialized cooperatives to regulate farmers' production practices. In terms of cooperative supervision, rewards and punishments should be appropriately increased, and a reward system should be established for high-quality agricultural products to encourage cooperatives to standardize and manage farmers' green production behaviors and urge farmers to take the initiative when applying green medicine and fertilizer (29). In terms of farmers, the government can appropriately increase the investment in the supervision cost of the quality and safety of agricultural products within a certain range, increase publicity efforts, use social forces to effectively spread the concept of green development in agriculture, enhance farmers' awareness of green production and improve their green production skills (16, 29, 38). The government provides discounts to farmers who purchase green pesticides and fertilizers, and the cooperative pays dividends to green farmers, which can greatly encourage farmers to adopt green production solve the quality and safety problems of agricultural products at the source, and prevent consumer health problems, and improve public health awareness.

As the middle player in the tripartite game, farmer-specialized cooperatives should actively respond to government decisions and fully mobilize the enthusiasm of farmers to realize a more comprehensive and standardized management of farmers' green production behaviors. According to Lewin's field theory, people's behaviors in the surrounding environment are affected by various environmental factors, and the cooperative management of farmers' production directly affects farmers' green production behaviors. At the same time, Nikolić et al. (39) believe that continuous education plays a key role in preventing pressure from safety experts, and it also plays a key role in the pressure management of the entire organization (39). Therefore, cooperatives should strengthen the management and guidance of farmers' production behaviors, and at the same time strengthen the continuous education of farmers' safety awareness, concepts, and attitudes to avoid problems such as excessive application of pesticides and fertilizers caused by farmers' lack of relevant knowledge. In addition, Jahangiri et al. (40) believe that establishing a high level of safety culture maturity can improve flexible engineering capabilities and effectively prevent the occurrence of safety accidents in the manufacturing industry (40). This method is also applicable to cooperatives engaged in agricultural production. The cooperative can manage the production of farmers by establishing a safe and complete management mechanism, so as to achieve the purpose of preventing the production of inferior agricultural products. Specific measures include the following: (1) Cooperatives regularly contact agricultural research institutions, organize professionals to provide practical green production technology guidance to farmers, and spread innovative technologies to rural areas. (2) Cooperatives appropriately increase dividends (punishments) paid (inflicted) to (on) green (nongreen) production farmers to encourage farmers to take the initiative in green production. (3) Cooperatives unify production standards and agricultural supplies, supplemented by human capital training and organizational culture construction; make management methods scientific, standardized and systematic; and construct a modern management mechanism of self-restraint, self-improvement and self-motivation.

Farmers directly affect the quality and safety of agricultural products. Irrational fertilization and drug application by farmers is a main factor leading to frequent agricultural product safety accidents. The existing literature shows that when farmers engage more in safe proactive behaviors, fewer safety accidents occur. Farmers should be aware of the importance of their own behaviors and the impact of their produce on the physical and mental health of consumers and actively respond to the government's policies and calls. Regarding the rules and regulations formulated by cooperatives, farmers should actively cooperate, carry out green production according to standards, provide high-quality agricultural products, actively participate in technical training organized by cooperatives, enhance their awareness of green production, reduce their dependence on traditional pesticides (41), effectively prevent the production of inferior agricultural products and the impact of inferior agricultural products on people's health and fundamentally solve the quality and safety problems of agricultural products.

This study identifies safe production measures in the government, farmer-specialized cooperatives and farmers evolutionary path and the evolutionary law of behavioral decision-making. It also uncovers the main decision-making mechanisms to achieve an ideal state of equilibrium and stability conditions and conduct data simulation experiments. Provide theoretical reference and practical guidance for government makes regulatory decisions, standardized management of cooperatives and implementation of Farmers' Green Production Behavior. Let farmers have more advanced awareness and technology in agricultural production, and ultimately achieve the purpose of eliminating the frequent occurrence of agricultural product quality and safety problems and improving public health. This research also has certain limitations. The next step is to expand the research scope by including agricultural enterprises and consumers to explore the characteristics and rules of multiagent behavioral decision-making regarding the quality and safety of agricultural products.

## Data availability statement

The original contributions presented in the study are included in the article/supplementary material, further inquiries can be directed to the corresponding author.

## Author contributions

YT and BP: conceptualization. YT: methodology, investigation, writing—review and editing, supervision, and funding acquisition. BP: software and data curation. JW, HY, and LM: validation. JW: formal analysis and writing—original draft preparation. ZT: resources. HY: visualization. LM: project administration. All authors have read and agreed to the published version of the manuscript.

## Funding

This study was funded by the National Social Science Fund of China - Research on multiple driving mechanism of black land conservation based on total quality management in Northeast China (Grant number 22BJY240).

## Conflict of interest

The authors declare that the research was conducted in the absence of any commercial or financial relationships that could be construed as a potential conflict of interest.

## Publisher's note

All claims expressed in this article are solely those of the authors and do not necessarily represent those of their affiliated organizations, or those of the publisher, the editors and the reviewers. Any product that may be evaluated in this article, or claim that may be made by its manufacturer, is not guaranteed or endorsed by the publisher.

## Appendix

$$\begin{array}{l} V_{1x}=app1yz\left(-\;C_{1}\;+\;D_{1}\;+\;W_{1}\;-\;M_{2}\right)\\ \;+\;y\left(1\;-\;z\right)\left(\;-\;C_{1}\;+\;D_{1}\;+\;W_{1}\;-\;M_{2}\right)\\ \;+\;z\left(1\;-\;y\right)\left(\;-\;C_{1}\;+\;W_{1}\;+\;M_{1}\right)\\ \;+\;\left(1\;-\;y\right)\left(1\;-\;z\right)\left(\;-\;C_{1}\;+\;W_{1}\;+\;M_{1}\right)\\ \;=\;y\left(D_{1}\;-\;M_{1}\;-\;M_{2}\right)\;+\;\left(\;-\;C_{1}\;+\;W_{1}\;+\;M_{1}\right) \end{array}$$

$$\begin{array}{l} V_{2x}\;=\;yz\left(C_{1}\;+\;D_{1}\;-\;W_{2}\right)\\ \;+\;y\left(1\;-\;z\right)\left(C_{1}\;+\;D_{1}\;-\;W_{2}\right)\\ \;+z\left(1\;-\;y\right)\left(C_{1}\;-\;D_{2}\;-\;W_{2}\right)\\ \;+\;\left(1\;-\;y\right)\left(1\;-\;z\right)\left(C_{1}\;-\;D_{2}\;-\;W_{2}\right)\\ \;=\;2yD_{1}\;+\;\left(C_{1}\;-\;D_{1}\;-\;W_{2}\right) \end{array}$$

$$\begin{array}{l} \bar{V_{x}}\;=\;x*V1x\;+\;\left(1\;-\;x\right)V2x\\ \;=x\left[y\left(D_{1}\;-\;M_{1}\;-\;M_{2}\right)\;+\;\left(\;-\;C_{1}\;+\;W_{1}\;+\;M_{1}\right)\right]\\ \;+\;\left(1\;-\;x\right)\left[2yD_{1}\;+\;\left(C_{1}\;-\;D_{1}\;-\;W_{2}\right)\right] \end{array}$$

$$\begin{array}{l} V_{1y}\;=\;xz\left(\;-\;C_{2}\;+\;M_{2}\;-\;M_{4}\;+\;D_{3}\;+\;B_{1}\right)\\ \;+x\left(1\;-\;z\right)\left(\;-\;C_{2}\;+\;M_{2}\;+\;M_{3}\;-\;D_{4}\;-\;B_{2}\right)\\ \;+\;z\left(1\;-\;x\right)\left(\;-\;C_{2}\;-\;M_{4}\;+\;D_{3}\;+\;B_{1}\right)\\ \;+\left(1\;-\;x\right)\left(1\;-\;z\right)\left(\;-\;C_{2}\;+\;M_{3}\;-\;D_{4}\;-\;B_{2}\right)\\ \;=\;xM_{2}\;+\;z\left(\;-\;C_{2}\;-\;M_{4}\;+\;D_{3}\;+\;B_{1}\right)\\ \;+\;\left(1\;-\;z\right)\left(\;-\;C_{2}\;+\;M_{3}\;-\;D_{4}\;-\;B_{2}\right) \end{array}$$

$$\begin{array}{l} \bar{V_{y}}\;=\;y•V1y\;+\;\left(1\;-\;y\right)V2y\\ \;=y[xM_{2}\;+\;z\left(\;-\;C_{2}\;-\;M_{4}\;+\;D_{3}\;+\;B_{1}\right)\\ \;+\left(1\;-\;z\right)\left(\;-\;C_{2}\;+\;M_{3}\;-\;D_{4}\;-\;B_{2}\right)\\ \;+\;\left(1\;-\;y\right)[\;-\;xM_{1}\;+\;C_{2}\;+\;z\left(D_{3}\;+\;B_{1}\right)\\ \;+\;\left(1\;-\;z\right)\left(-\;D_{4}\;-\;B_{2}\right)] \end{array}$$

$$\begin{array}{l} V_{1z}=\;xy\left(T_{1}\;-\;C_{3}\;+\;S_{1}\;+\;M_{4}\;+\;W_{3}\right)\\ \;+\;x\left(1\;-\;y\right)\left(T_{1}\;-\;C_{3}\;+\;S_{1}\;+\;W_{3}\right)\\ \;+\;y\left(1\;-\;x\right)\left(\;-\;T_{2}\;-\;C_{3}\;+\;S_{1}\;+\;M_{4}\;+\;W_{3}\right)\\ \;+\;\left(1\;-\;x\right)\left(1\;-\;y\right)\left(\;-\;T_{2}\;-\;C_{3}\;+\;S_{1}\;+\;W_{3}\right)\\ \;=xT_{1}\;-\;\left(1\;-\;x\right)T_{2}\;+\;yM_{4}\;+\;\left(-\;C_{3}\;+\;S_{1}\;+\;W_{3}\right) \end{array}$$

$$\begin{array}{l} V_{2z}\;=\;xy\left(T_{1}\;+\;C_{3}\;-\;S_{2}\;-\;M_{3}\;-\;W_{4}\right)\\ \;+\;x\left(1\;-\;y\right)\left(T_{1}\;+\;C_{3}\;-\;S_{2}\;-\;W_{4}\right)\\ \;+\;y\left(1\;-\;x\right)\left(\;-\;T_{2}\;+\;C_{3}\;-\;S_{2}\;-\;M_{3}\;-\;W_{4}\right)\\ \;+\;\left(1\;-\;x\right)\left(1\;-\;y\right)\left(\;-\;T_{2}\;+\;C_{3}\;-\;S_{2}\;-\;W_{4}\right)\\ \;=\;xT_{1}\;-\;\left(1\;-\;x\right)T_{2}\;-\;yM_{3}\;+\;\left(C_{3}\;-\;S_{2}\;-\;W_{4}\right) \end{array}$$

## References

[1] Wang J Liu Z Li Q. Risk management of agricultural products: A case study of pesticide application in agricultural production. Chin Rural Econ. (2015) 31:54–62 + 76.26855353

[2] Nowacki K. The impact of implemented management systems on the safety culture of work in production. Multidiscip Asp Prod Eng. (2019) 2:243–52. 10.2478/mape-2019-0024

[3] Zhang M Zeiss MR Geng S. Agricultural pesticide use and food safety: California's model. J Integr Agric. (2015) 14:2340–57. 10.1016/S2095-3119(15)61126-1

[4] Ping H Wang J Ma Z. Du, Y. Mini-review of application of IoT technology in monitoring agricultural products quality and safety. Int J Agric Biol Eng. (2018) 11:35–45. 10.25165/ijabe.v11i5.3092

[5] Vidergar P Perc M Lukman K. A survey of the life cycle assessment of food supply chains. J Clean Prod. (2020) 286:125506. 10.1016/j.jclepro.2020.125506

[6] Akande MG Sanni FS Enefe NG. Human Health Risk Evaluation of Organophosphate Insecticide Residues in Post-Harvest Cowpea in Gwagwalada, Abuja, Nigeria. J Health Pollut. (2020) 10:201203. 10.5696/2156-9614-10.28.201203 33324500 PMC7731488

[7] Deng M Xiang G Yao S. The effectiveness of the multilateral coalition to develop a green agricultural products market in China based on a TU cooperative game analysis. Sustainability. (2018) 10:1476. 10.3390/su10051476

[8] Yan W Zhang Z Zhang Q Zhang G Hua Q Li Q. Deep data analysis-based agricultural products management for smart public healthcare. Front Public Health. (2022) 10:847252. 10.3389/fpubh.2022.847252 35462816 PMC9021602

[9] Jallow MFA Awadh DG Albaho MS Devi VY. Thomas BM. Pesticide risk behaviors and factors influencing pesticide use among farmers in Kuwait. Sci Total Environ. (2017) 574:490–8. 10.1016/j.scitotenv.2016.09.085 27644027

[10] Fan L Niu H Yang X Qin W Bento CP Ritsema CJ. Factors affecting farmers' behaviour in pesticide use: Insights from a field study in northern China. Sci Total Environ. (2015) 537:360–8. 10.1016/j.scitotenv.2015.07.150 26282770

[11] Yin W Fu G Yang C Jiang Z Zhu K Gao Y. Fatal gas explosion accidents on Chinese coal mines and the characteristics of unsafe behaviors: 2000–2014. Saf Sci. (2017) 92:173–9. 10.1016/j.ssci.2016.09.018

[12] Liu EM Huang JK. Risk preferences and pesticide use by cotton farmers in China. J Dev Econ. (2013) 103:202–15. 10.1016/j.jdeveco.2012.12.005

[13] Zhao L Wang C Gu H Yue C. Market incentive, government regulation and the behavior of pesticide application of vegetable farmers in China. Food Control. (2018) 85:308–17. 10.1016/j.foodcont.2017.09.016

[14] Ngowi AVF Mbise TJ Ijani ASM London L Ajayi OC. Smallholder vegetable farmers in Northern Tanzania: Pesticides use practices, perceptions, cost and health effects. Crop Prot. (2007) 26:1617–24. 10.1016/j.cropro.2007.01.008 18528532 PMC2410092

[15] Arabian A Omidi L Bakhshi E Ghanbari A Torabinassaj E Zakerian SA. Assessment of occupational safety, health, and ergonomics issues in agriculture in some cities of Iran. Work. (2020) 65:89–96. 10.3233/WOR-193061 31868715

[16] Zhang L Li X Yu J Yao X. Toward cleaner production: what drives farmers to adopt eco-friendly agricultural production? J Clean Prod. (2018) 184:550–8. 10.1016/j.jclepro.2018.02.272

[17] Bhandari G Zomer P Atreya K Mol HG Yang X Geissen V. Pesticide residues in Nepalese vegetables and potential health risks. Environ Res. (2019) 172:511–21. 10.1016/j.envres.2019.03.002 30852454

[18] Grewal AS. Pesticide Res-idues in Food Grains, Vegetables and Fruits: a hazard to human health. J Med Chem Toxicol. (2017) 6:2.

[19] Charlier C Valceschini E. Coordination for traceability in the food chain. A critical appraisal of European regulation. Eur J Law Econ. (2008) 25:1–15. 10.1007/s10657-007-9038-2

[20] Langiano E Ferrara M Lanni L Viscardi V Abbatecola AM De Vito E. Food safety at home: knowledge and practices of consumers. J Public Health. (2012) 20:47–57. 10.1007/s10389-011-0437-z 22347771 PMC3268974

[21] Zhou J Jin S. Adoption of Food Safety and Quality Standards by China's Agricultural Cooperatives: A Way out of Monitoring Production Practices of Numerous Small-Scale Farmers? International Association of Agricultural Economists. International Association of Agricultural Economists (2009).

[22] Gu LL Guo QH. The Analysis on function and operating mechanism of farmer cooperatives in the process of safety management. China Bus Mark. (2015) 29:100–5. 10.14089/j.cnki.cn11-3664/f.2015.08.016

[23] Jin S Zhou J. Adoption of food safety and quality standards by China's agricultural cooperatives. Food Control. (2011) 22:204–8. 10.1016/j.foodcont.2010.06.021

[24] Bijman J. Agricultural Cooperatives and Market Orientation: A Challenging Combination? Routledge. (2016). p. 151–68.

[25] Ito J Bao Z Su Q. Distributional effects of agricultural cooperatives in China: Exclusion of smallholders and potential gains on participation. Food Policy. (2012) 37:700–9. 10.1016/j.foodpol.2012.07.009

[26] Bijman J Hu D. The rise of new farmer cooperatives in China; Evidence from Hubei Province. J Rural Coop. (2011) 39:99–113. 10.22004/AG.ECON.114252

[27] Deng H Huang J Xu Z Rozelle S. Policy support and emerging farmer professional cooperatives in rural China. China Econ Rev. (2010) 21:495–507. 10.1016/j.chieco.2010.04.009

[28] Chen XJ Tan YW. Study on influencing factors of services functions of farmers cooperatives from the perspective of food safety-the case of fruits cooperatives in Guangdong Province. J Agrotech Econ. (2013) 1:121–8. 10.13246/j.cnki.jae.2013.01.014

[29] Cui H Zhao T Tao P. Evolutionary game study on the development of green agriculture in china based on ambidexterity theory perspective. Polish J Environ Stud. (2019) 28:1093–104. 10.15244/pjoes/87139

[30] Dang H. Tripartite game model of agricultural product supply Chain based on evolutionary game. In: 2018 8th International Conference on Logistics, Informatics and Service Sciences (LISS). Beijing: IEEE (2018). p. 1-5.

[31] Liu L Zhu Y Guo S. The evolutionary game analysis of multiple stakeholders in the low-carbon agricultural innovation diffusion. Complexity. (2020) 2020:1–12. 10.1155/2020/6309545

[32] Du J Zhou Z Xu L. Evolutionary game mechanism on complex networks of green agricultural production under intensive management pattern. Complexity. (2020) 2020:1–13. 10.1155/2020/8541517

[33] Xu L Zhou Z Du J. An evolutionary game model for the multi-agent co-governance of agricultural non-point source pollution control under intensive management pattern in China. Int J Environ Res Public Health. (2020) 17:2472. 10.3390/ijerph17072472 32260432 PMC7177998

[34] Li C Shi Y Khan SU Zhao M. Research on the impact of agricultural green production on farmers' technical efficiency: evidence from China. Environ Sci Pollut Res. (2021) 28:1–17. 10.1007/s11356-021-13417-4 33735411

[35] Adnan N Nordin SM Bahruddin MA Tareq AH. A state-of-the-art review on facilitating sustainable agriculture through green fertilizer technology adoption: assessing farmers behavior. Trends Food Sci Technol. (2019) 86:439–52. 10.1016/j.tifs.2019.02.040

[36] Sandholm WH. Evolutionary game theory. Complex Social and Behavioral Systems: Game Theory and Agent-Based Models. Berlin: Springer Nature (2020). p. 573-608.

[37] McGill BJ Brown JS. Evolutionary game theory and adaptive dynamics of continuous traits. Annu Rev Ecol Evol Syst. (2007) 38:403–35. 10.1146/annurev.ecolsys.36.091704.175517

[38] Hald KS. Social influence and safe behavior in manufacturing. Saf Sci. (2018) 109:1–11. 10.1016/j.ssci.2018.05.00833318892

[39] Nikolić V Taradi J Petković AI. Workplace stress of occupational safety specialists in Croatia and Serbia. Work. (2021) 70:419–31. 10.3233/WOR-213581 34633344

[40] Jahangiri M Zinat-Motlagh K Ghaem H Zinat-Motlagh F Kamalinia M Banaee S. Safety culture maturity and resilience engineering in an oil drilling industry: A comparison study among government-owned and private companies. Work. (2021) 70:443–53. 10.3233/WOR-213583 34633346

[41] Borovik VS Borovik VV Skorobogatchenko D. Model of the strategy for reducing the road accident rate in the city. Transp Res Procedia. (2018) 36:68–76. 10.1016/j.trpro.2018.12.045

